# Prevalence and Antimicrobial Resistance Patterns of *Salmonella* in Asymptomatic Horses in Eastern Spain: A One Health Perspective

**DOI:** 10.3390/ani15233413

**Published:** 2025-11-26

**Authors:** María Socorro Simó-Martínez, Ana Marco-Fuertes, Ángela Galán-Relaño, Rafael J. Astorga Márquez, Clara Marin, Antonio Valero Díaz, Santiago Vega

**Affiliations:** 1 Department of Animal Medicine and Surgery, Universidad Cardenal Herrera—CEU, CEU Universities, 46115 Valencia, Spain; 2 Department of Animal Production and Health, Public Veterinary Health and Food Science and Technology, Faculty of Veterinary Medicine, Universidad Cardenal Herrera—CEU, CEU Universities, 46115 Valencia, Spain; ana.marcofuertes@uchceu.es (A.M.-F.); svega@uchceu.es (S.V.); 3 Department of Nursing, Pharmacology and Physiotherapy Area, Faculty of Veterinary Medicine, University of Cordoba, 14014 Cordoba, Spain; 4 Animal Health Department, Veterinary Faculty, University of Cordoba, 14014 Cordoba, Spain; sa1asmar@uco.es; 5 Food Science and Technology Department, Veterinary Faculty, University of Cordoba, 14014 Cordoba, Spain

**Keywords:** *Salmonella*, antimicrobial resistance, horses, prevalence, One Health

## Abstract

**(1) Background:** *Salmonella* are zoonotic pathogens, and rising antimicrobial resistance (AMR) amplifies their public health impact. Asymptomatic horses can act as reservoirs, contributing to environmental contamination and interspecies transmission. This study aimed to estimate the prevalence of *Salmonella* and characterize AMR patterns in healthy horses from eastern Spain. **(2) Methods:** Faecal samples from 95 asymptomatic horses were collected once daily over five consecutive days (475 samples in total) and processed under for *Salmonella* detection. Epidemiological information was obtained through owner questionnaires, and associations with *Salmonella* shedding were analyzed using generalized linear models. Antimicrobial susceptibility was assessed by minimum inhibitory concentration assays following the European Committee on Antimicrobial Susceptibility Testing (EUCAST) criteria. **(3) Results:** *Salmonella* was detected in 25.3% of horses (24/95), with S. Enteritidis, S. Johannesburg, and S. Virchow as the most frequent serotypes. A significant association was observed between proximity of manure storage and bacterial detection (*p* < 0.001). Among 24 isolates of *Salmonella*, 88.9% were resistant to at least one antimicrobial, and 50% exhibited multidrug resistance. The highest resistance rates were against sulfamethoxazole and gentamicin, followed by ciprofloxacin and tigecycline. **(4) Conclusions:** Healthy horses can act as silent carriers of multidrug-resistant *Salmonella*, highlighting the need for surveillance, strengthened biosecurity, and prudent antimicrobial use within a One Health framework.

## Simple Summary

Horses can carry bacteria that may be harmful to both animals and humans, even when they show no signs of illness. One of these bacteria, *Salmonella enterica*, can spread through the environment and contribute to infections that are difficult to treat because of growing resistance to antibiotics. This study examined healthy horses in eastern Spain to find out how common this bacterium is and whether it shows resistance to antibiotics. Faecal samples from 95 horses were collected once daily over five consecutive days (475 samples in total), and *Salmonella* was detected in approximately one quarter of the animals. The most frequent types detected are known to cause disease in humans. Many of these bacterial strains were resistant to one or more antibiotics, and half were resistant to several at the same time. The study also found that poor hygiene and the storage of manure close to the animals were related to higher bacterial presence. These results show that healthy horses can act as silent carriers of bacteria that are important for public health. Monitoring them regularly and improving hygiene in equine facilities could help reduce the spread of antibiotic-resistant bacteria and protect both animal and human health.

## 1. Introduction

Throughout history and the domestication process of animals, horses and humans have shared a close and enduring relationship. Initially, horses were primarily used as a means of transportation, labour, and food production. However, Spain’s equine industry, one of the oldest globally, has undergone significant transformation in recent decades, marked by a notable shift in its economic focus. The sector has progressively diversified from traditional functions toward activities related to leisure, sports, tourism or genetics improvement [1].

The close human-horse relationship and the shared environment create a potential risk of transmission of zoonotic diseases. Among these, salmonellosis of public health importance. In horses, *Salmonella* infection may be subclinical or manifest with clinical signs ranging from mild symptoms such as fever and dehydration to diarrhoea, colic, and manifestations of septicaemia [2,3,4,5]. Moreover, horses can act as reservoirs, shedding the bacterium into the environment and facilitating transmission to humans and other animals. In fact, *Salmonella* spp. is one of the leading pathogens causing foodborne infections in humans across Europe, with over 77,486 confirmed cases in 2023 [6]. It is estimated that approximately 150 million people worldwide become ill with non-typhoidal *Salmonella* (NTS) each year, resulting in around 60,000 deaths [7].

Equine salmonellosis may occur as sporadic cases or as outbreaks [8,9,10,11,12,13,14,15,16,17,18], including in veterinary hospitals, where mortality rates have reach up to 44% [8,9,10,12,14,19,20,21]. Hospitalization and antimicrobial therapy have been shown to increase horses’ susceptibility to *Salmonella* infection [19], particularly to serotypes shed by asymptomatic horses present in same facilities [5,14]. This underscores the importance of controlling environmental sources of contamination in equine settings.

Beyond this pathogenic potential, *Salmonella* spp. is one of the most critical pathogens according to the World Health Organization (WHO) due to its high levels of antimicrobial resistance (AMR) [22]. Consequently, horses may act not only as disseminators of this bacterium, but also as carriers of the AMR genes. Indeed, the WHO classifies AMR as one of the major challenges facing global public health, primarily due to the emergence of multidrug-resistant (MDR) strains, which complicate treatment and reduce therapeutic efficacy. Overall, both directly and indirectly, AMR causes 4.95 million deaths each year [22] making it imperative to evaluate and monitor AMR in this bacterium. One of the main reasons for the increasing rate of salmonellosis outbreaks is the presence of MDR strains of *Salmonella* [8,11,16,23,24], which complicate treatment and lead to therapeutic failures. Therefore, understanding the role of horses as potential disseminators of pathogens and AMR genes within shared environments is essential for developing effective prevention strategies under the One Health approach.

In this context, the main objective of the present study was to assess the prevalence of *Salmonella* spp. in asymptomatic horses and to evaluate the influence of external risk factors on its presence. Additionally, the antimicrobial resistance (AMR) and multidrug resistance (MDR) profiles of isolates recovered from horses in the *Hoya de Buñol* geographic area (Valencia Region, Spain) were characterized.

## 2. Materials and Methods

### 2.1. Experimental Design

To determine the prevalence of *Salmonella* spp. in asymptomatic horses, sampling was conducted in the *Hoya de Buñol* region, geographic area located within the Province of Valencia (Eastern Spain). This area was selected as a representative subset of the healthy equine population, given its demographic and territorial characteristics, which reflect those of the broader horse population in the Valencian Community. Since no previous studies had investigated the prevalence using a sampling protocol that accounted for the intermittent shedding of the bacterium, samples were obtained from a representative number of animals to ensure a reliable estimation of carrier status within the population.

The animal study was reviewed and approved by the Animal Ethics Committee at UCH-CEU University (code of research CEEA 22/05).

Written informed consent was obtained from all horse owners prior to sampling and data collection.

### 2.2. Epidemiological Data Collection

Along with sample collection, a structured questionnaire was completed by the horse owners, gathering general information about each animal as following: sex, age or breed, housing conditions, and lifestyle. Additional data collected through the epidemiological survey included pathology history, recent pharmacological treatments (e.g., antibiotics, anti-inflammatories, or other drugs) and horse’s primary activity, which was grouped into three major categories: leisure, sport, and breeding/reproduction. This questionnaire is provided in the Supplementary Materials (Supplementary Materials S1).

For statistical analysis, horses were classified by age into three categories: foals/young horses (0 to 5 years old), adults (6 to 18 years old) and geriatrics (>19 years old) [25,26,27,28].

Housing types were categorized as stall, paddock, or a combination of both. Information was compiled regarding the cleanliness of the facilities, including how often manure was cleaned and the distance of the manure storage from the animals. The cleaning frequency of the stalls/paddocks was categorized as daily, weekly, monthly/or less frequent. Additionally, the distance from the housing to the manure storage was considered and grouped into three categories: <10 m, 11–99 m, and >100 m.

The sampled horses resided in either riding schools or breeding farms, and included individuals housed in stalls, outdoor paddocks, or both. Horses living individually as well as those cohabiting with other horses were included in the study.

### 2.3. Sample Collection

In the absence of epidemiological studies on the prevalence of *Salmonella* in asymptomatic horses in Europe, an estimate of the prevalence was determined through the random sampling of 100 horses from a specific area, *Hoya de Buñol*, which has been determined to have a population size of 539 horses.

Prior to sampling, each horse underwent a clinical examination, and vital parameters were recorded to confirm the absence of fever or diarrhea. Faecal samples were collected from the rectal ampulla of each horse over five consecutive days, given the intermittent shedding of the bacterium.

All samples were transported under refrigeration at ≤4 °C to the Veterinary Sciences Faculty microbiology laboratory from the University CEU Cardenal Herrera. Microbiological analyses were performed on the same day as the sample collection.

### 2.4. Salmonella Isolation

For the isolation of *Salmonella* spp., all samples were processed in accordance with ISO 6579-1:2017 [29]. Briefly, faecal samples were pre-enriched in buffered peptone water (BPW; Scharlau^®^, Barcelona, Spain) at a ratio of 1:10 *v*/*v* and were then incubated at 37 ± 1 °C for 24 h. Subsequently, 0.1 mL of the pre-enriched culture was inoculated onto a Modified Rappaport Vassiliadis agar plate (MSRV; Marcy l’Etoile, France) and incubated at 41.5 ± 1 °C for 48 h. Positive MSRV plates were subsequently transferred to Xylose Lysine Deoxycholate agar (XLD, Scharlau^®^, Barcelona, Spain) and a selective chromogenic agar (ASAP; Marcy l’Étoile, France), followed by incubation at 37 ± 1 °C for 24–48 h.

Confirmed isolates were serotyped according to the Kauffmann–White scheme [30] at the National Reference Laboratory for Animal Health (Algete, Madrid, Spain) and stored at −80 °C for further analyses.

### 2.5. Antimicrobial Susceptibility Testing

Antimicrobial susceptibility tests were performed using the EU Surveillance *Salmonella*/*E. coli* EUVSEC3 Sensititre Plate (Thermo Scientific™Sensititre™, Madrid, Spain). This plate includes 15 antimicrobials of public health relevance and complies with the monitoring and reporting of antimicrobial resistance in zoonotic and commensal bacteria in food-producing animals, as outlined in Decision (EU) 2020/1729 [31].

The AMR was evaluated using the minimum Inhibitory concentration (MIC) assay with Thermo Scientific™ Sensititre™ Plates (Madrid, Spain) according to the manufacturer’s instructions. All results were interpreted based on the clinical breakpoints established by the European Committee on Antimicrobial Susceptibility Testing (EUCAST) in 2024 [32]. The antimicrobials tested, and their corresponding concentration ranges are detailed in Supplementary Materials S2 (Table S1). Additionally, multidrug resistance (MDR) was defined as acquired resistance to at least one agent within three or more antibiotic classes [33].

For antimicrobial susceptibility testing, frozen *Salmonella* isolates were thawed and cultured on nutrient agar, followed by incubation at 37 ± 1 °C for 24 h. After incubation, individual colonies were suspended in 5 mL of sterile demineralized water (T3339; ThermoFisher Scientific™, Madrid, Spain). Each bacterial suspension was mixed and standardized to a density of 0.5 McFarland using a Nephelometer (ThermoFisher Scientific™, Madrid, Spain). Subsequently, 10 μL of the suspension were inoculated in a vial containing 11 mL of Mueller–Hinton broth (T3462; ThermoFisher Scientific™, Madrid, Spain) and mixed. From this mixture, 50 μL of the vial contents were transferred into each well of the Sensititre plate. The plates were then sealed with adhesive film and incubated at 37 ± 1 °C for 24 h. Manual reading of the plates was carried out using a Sensititre Vizion (Thermo Scientific™ Sensititre™ Vizion™ Digital MIC Viewing System, ThermoFisher Scientific, Madrid, Spain).

### 2.6. Statistical Analysis

A binomial generalized linear model (GLM) with a probit link was fitted to assess associations between *Salmonella* spp. carriage and the evaluated epidemiological risk factors (age, sex, medical history including prior pathologies and treatments, equine fitness level, recent travel, housing type, distance from the manure storage area, sanitation frequency, and direct interspecies contact). The same model structure was used to explore associations between these factors and AMR or MDR among *Salmonella* spp. isolates. Groupwise differences in proportions were further assessed using Pearson’s χ^2^ tests, with Fisher’s exact tests applied whenever any expected cell count was <5, to corroborate GLM findings. Statistical significance was set at α = 0.05 (two-tailed). Data are presented as estimated marginal means (EMMs) ± standard errors. Analyses were performed in R 4.4.2 (R Core Team, Vienna, Austria) using the emmeans, car, and multcompView packages.

Pairwise comparisons between factor levels were derived from estimated marginal means (emmeans) with Sidak adjustment for multiple testing; adjusted *p*-values and 95% confidence intervals are reported.

## 3. Results

### 3.1. Epidemiological Data

A total of 95 asymptomatic horses were sampled. Of the 100 equines initially earmarked for sampling, five had to be excluded from the study, as samples could not be obtained from them for five consecutive days.

From these 95 asymptomatic horses, 31.6% corresponded to geldings (30/95), 37.9% mares (36/95) and 30.5% stallions (29/95), age ranging from 3 months to 30 years. According to the epidemiological survey completed by the owners, 10.5% (10/95) of the horses had a recorded history of previous pathology, and some had recently received pharmacological treatments, including antibiotics, anti-inflammatory drugs, or other medications. Regarding their primary activity, 64.2% (61/95) were classified as leisure horses, 30.5% (29/95) as sport horses, and 5.3% (5/95) as breeding/reproductive animals.

**Figure 1:**
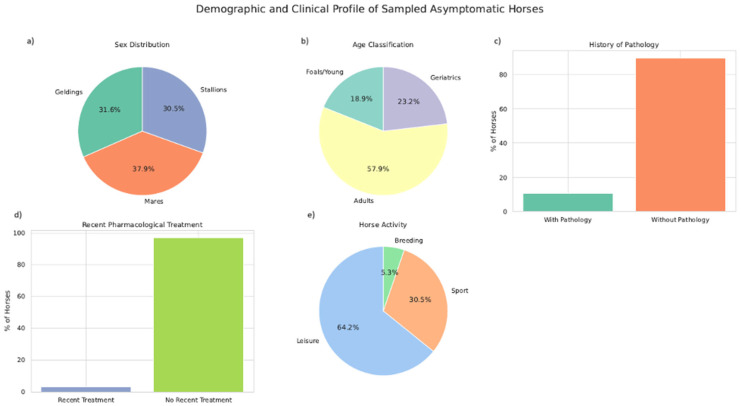
Demographic and clinical characteristics of the 95 asymptomatic horses included in the study. (**a**) Sex distribution, (**b**) Age classification, (**c**) History of pathology, (**d**) Recent pharmacological treatments, and (**e**) Main activity of the horses.

All these demographic and clinical characteristics are represented in Figure 1.

**Figure 2:**
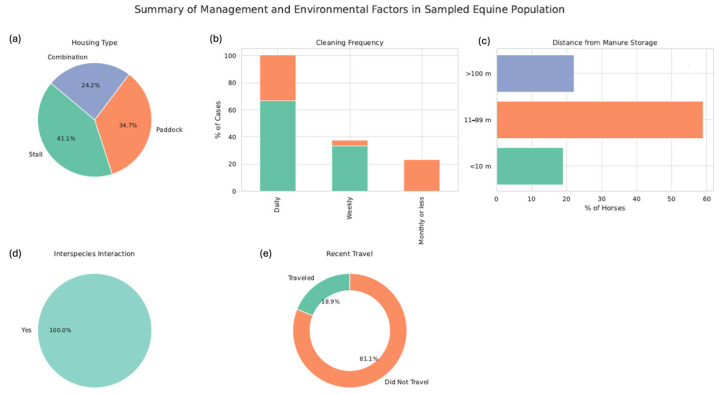
Management and environmental characteristics of the sampled equine population. (**a**) Distribution of housing systems among horses, categorized as stalls, paddocks, or a combination of both. (**b**) Cleaning frequency of housing facilities, represented as a percentage of horses living under each system. (**c**) Distance between horse accommodations and manure storage sites. (**d**) Proportion of horses with regular interspecies interaction, including contact with conspecifics, other domestic animals, or wildlife. (**e**) Percentage of horses that had recently travelled for purposes such as competitions, fairs, or excursions. These variables are relevant as potential risk factors for the presence and spread of antimicrobial-resistant *Salmonella*.

Additional information collected through the surveys concerned housing conditions, sanitation practices, interspecies interactions, and animal mobility. Most horses were housed in stalls (41.1%), with 66.6% of these cleaned daily. However, for paddocks, cleaning was performed daily in only 33.7% of cases. Regarding the distance between the horse accommodations and the manure storage, it was obtained that 18.9% of the horses were housed less than 10 m from the storage, 58.9% were between 11 and 99 m, and the remaining 22.1% were more than 100 m away from the manure storage. Furthermore, 18.9% of the sampled equine population had recently travelled for various purposes, including competitions, excursions and fairs (Figure 2).

### 3.2. Salmonella Identification and Serotyping

In total, 95 asymptomatic horses over five consecutive days were sampled, resulting in 475 samples collected. Out of the 95 horses sampled, 24 resulted positives to *Salmonella* spp. (25.3%) in at least one sample, with a total of 39 positive samples. Most positives were isolated to a single day; however, intermittent or sustained shedding was also observed. Specifically, three horses tested positive on two consecutive days, five on two non-consecutive days, two on three consecutive days, and one on four of five days separated by a single negative day (Table 1).

**Figure 3:**
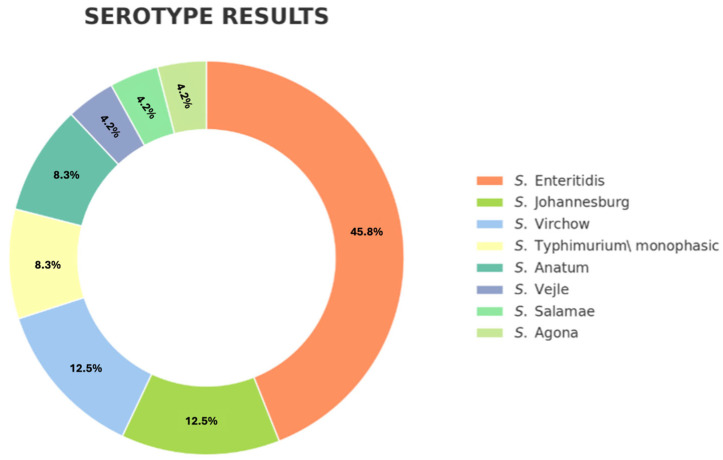
Graphical representation of the serotyping results for *Salmonella* spp. isolated from equine faecal samples.

All *Salmonella* isolates were identified as *Salmonella* enterica subsp. enterica (n = 39). Regarding serotyping, only one isolate from each horse was submitted for serotyping, resulting in a total of 24 strains analyzed. The main serotypes isolated were S. Enteritidis at 45.8% (11/24), followed by S. Johannesburg and S. Virchow both 12.5% (3/24). Additionally, S. Typhimurium monophasic Variant (mST) and S. Anatum were present at 8.3% (2/24) (Figure 3).

### 3.3. Epidemiological Surveys

Subsequently, a statistical analysis was performed using the variables gathered from the epidemiological surveys to explore potential correlations between these factors and the presence of *Salmonella*-positive animals. The results showed a significant correlation between recent pharmacological treatments and the detection of *Salmonella* (*p* < 0.001). Among the treated horses, the prevalence of the *Salmonella* was 0% (0/3), compared with 26.08% (24/92) observed in the untreated group. While a discernible difference is evident, it should be interpreted with caution due to the marked imbalance in group sizes. Moreover, none of the treated horses had received antibiotics; the administered drugs belonged exclusively to the non-steroidal anti-inflammatory drugs (NSAID) group (phenylbutazone, flunixin meglumine). This inherent limitation of the sample suggests that, while an association is suggested, further research is required to validate and confirm this finding. Such research should comprise a more balanced sample distribution (Table 2).

In addition, significant associations were also identified between *Salmonella* detection and manure storage and paddock cleaning frequency. Horses housed in facilities where manure was stored within 10 m showed a markedly higher prevalence of *Salmonella* (50%; 9/18) compared to 25.42% (15/59) when manure was housed 11 and 99 m away, and 0% (0/21) when housed at distances greater than 100 m away (*p* < 0.001). Pairwise comparisons revealed that animals located more than 100 m from the manure storage site differed significantly from those located 11–99 m and those within 10 m; whereas no significant difference was detected between the 11–99 m and <10 m groups. Likewise, paddock cleaning frequency was also significantly associated with bacterial detection (*p* < 0.001). The prevalence reached 100% (4/4) in facilities with weekly cleaning, compared with 18.18% (4/22) with monthly cleaning and 28.12% (9/32) with daily cleaning. Pairwise analysis indicated that animals housed in paddocks cleaned on a weekly basis differed significantly from those in paddocks cleaned daily or monthly, while no significant difference was found between the daily and monthly cleaning groups. These findings emphasize the critical role of environmental management practices, particularly waste storage, as key factors shaping the *Salmonella* circulation in equine facilities (Table 2).

### 3.4. Antimicrobial Susceptibility Profiles

Among the isolated strains, it was found that 88.9% (21/24) exhibited resistance to at least one of the 15 antimicrobials tested, with 50% (12/24) of these strains showing a MDR pattern.

The highest resistance rates among the *Salmonella* isolates were observed for sulfamethoxazole and gentamicin, with 70.8% (17/24) and 42.9% (10/24) of isolates showing resistance, respectively. These were followed by ciprofloxacin 25% (6/24) (Table 3).

The complete distribution of antimicrobial resistance percentages for all tested antibiotics is presented in Table 4.

## 4. Discussion

*Salmonella* is a zoonotic pathogen of significant importance in both veterinary and public health. While often asymptomatic in horses, its presence poses a potential risk for transmission to humans, particularly in equestrian environments with frequent human–animal interaction. Monitoring healthy equine populations is essential for understanding the epidemiological patterns of *Salmonella*, detecting emerging serotypes, and identifying antimicrobial resistance profiles. Horses may act as silent reservoirs, contributing to environmental contamination and cross-species transmission. Consequently, surveillance efforts—particularly in regions with high equine density—are vital for early detection and for guiding antimicrobial stewardship within a One Health framework.

There are currently no studies reporting the prevalence of *Salmonella* in horses in Europe. One study on the presence of *Salmonella* and Shiga toxin-producing *E. coli* in horse faeces was conducted in Germany, analyzing faecal samples from 400 horses. PCR analyses were performed to detect *S. enterica*, and no positive samples were found [34]. In contrast, studies from the United States have shown that the prevalence of *Salmonella* shedding in horses can vary considerably depending on health status, season, and geographical region. Among healthy horses in the general equine population, the shedding prevalence is estimated at 0.8% [35] whereas in hospitalized horses, reported values range from 0.5% to 7%, depending on whether animals were tested upon admission or during hospitalization, respectively [2,10,17,36,37,38,39,40].

Detecting *Salmonella* in horses can be a diagnostic challenge due to the intermittent nature of bacterial shedding and the low concentration in equine faeces [21]. Consequently, regardless of the analytical method selected for *Salmonella* spp. detection and its sensitivity, the probability of detection from a single sample remains low. Additionally, because *Salmonella* is shed intermittently, as mentioned above, multiple consecutive samples from the same animal are recommended to improve diagnostic accuracy [14,41]. In the present study of asymptomatic horses, faecal samples were collected for five consecutive days, which likely explains the relatively high prevalence observed (25.3%). Notably, several *Salmonella*-positive horses were only identified on the fourth or even the fifth day of sampling (Table 1), supporting the enhanced sensitivity of extended sampling protocols for identifying intermittent shedders. Current recommendations are to take 3 to 5 samples at intervals of 12 to 24 h [17,21,42]. When interpreted in parallel, the estimated diagnostic sensitivity increases from 44% for a single sample, 66% for two, 82% for three, and 97% for five [21,42]. These findings underscore the importance of rigorous sampling protocols to avoid underestimating the true prevalence of *Salmonella* in equine populations.

Thus, differences in prevalence reported in U.S. studies may be explained by the fact that only a single sample per horse was typically collected [10,35,38]. Additionally, the results can vary depending on the isolation protocol used, as studies in other species indicate that the sample size/weight affects the success of isolating this bacterium [41].

In the present study, no significant associations were observed between the clinical variables collected and the presence of *Salmonella*. However, other studies have identified risk factors for the development of salmonellosis in horses, including feed restrictions and dietary changes [10,12]. Moreover, it has been demonstrated that elevated stress levels increased the risk of infection. This is attributed, for example, to transport and heat-induced stress, which may promote bacterial shedding in carrier animals and facilitate infection in susceptible animals [12,13,43]. Furthermore, foals are particularly vulnerable to infection due to increased exposure, reduced immunocompetence, and an undeveloped mature gut microbiota [14]. Other risk factors associated with increased risk include antibiotic therapy [14,44], abdominal surgery, gastrointestinal disease, and colic episodes [14,38], as these conditions may disrupt the intestinal microbiota and facilitate pathogen colonization [45]. In contrast to earlier findings, none of the aforementioned factors seemed to be associated with *Salmonella* shedding in the present study. Only environmental variables related to the manure storage distance and paddock cleaning frequency were significantly associated with *Salmonella* detection. The positive association between a greater distance from the manure storage area and a lower likelihood of *Salmonella* shedding seems reasonable, as horses are susceptible to contact with contaminated faeces, which facilitates infection. Interestingly, no statistically significant association was observed with the frequency of stall cleaning, whereas paddock cleaning frequency did show a significant association. Pairwise comparisons indicated differences between the weekly cleaning group and both the daily and monthly cleaning groups, while no difference was observed between the latter two. However, this finding may mainly because of only four horses were housed in paddocks with weekly cleaning, and all of them tested positive, in contrast to the larger sample sizes in the daily and monthly groups, which yielded more variable results. Consequently, this apparent significance should be interpreted with caution, as the sample size was not statistically representative. A statistically significant correlation was also found among horses receiving medical treatment; however, given the very limited number of treated animals, this result must likewise be interpreted with caution.

Geographic region may also influence shedding patterns with higher prevalence rates reported in warmer and more humid regions of North America compared to cooler and drier regions [35]. The horses included in our study were in the interior region of the eastern Iberian Peninsula, which is characterized by a Mediterranean-continental climate. Summers are typically short, warm, and mostly clear, while winters are long, cold, windy, and partly cloudy, with generally dry conditions throughout the year. Average annual temperatures typically range from 3 °C to 31 °C and rarely drop below −2 °C or rise above 34 °C [46]. However, these conditions are progressively changing caused by climate evolution [47]. Sampling in the present study was conducted between February and July. This climatic context should be considered when interpreting the observed prevalence, as higher *Salmonella* shedding rates would be expected in more humid regions.

*Salmonella enterica* subsp. *enterica* (subspecies I) is the most prevalent subspecies, accounting for approximately 99% of *Salmonella* infections in humans and other warm-blooded animals [19,48]. Among the *Salmonella* serotypes isolated in this study, *S*. Enteritidis, *S*. Virchow, and monophasic *S*. Typhimurium were identified. These serotypes are highly pathogenic to humans [20] and are recognized as major contributors to severe clinical outcomes, thus representing a significant public health concern. Beyond their virulence, these serovars are of particular concern due to their high AMR [22].

*S.* Enteritidis is considered a priority serotype in epidemiological surveillance, as it is the leading cause of human and animal clinical salmonellosis worldwide [18].

Monophasic *S.* Typhimurium is classified as an emerging serovar due to its increasing AMR; and *S.* Virchow has been designated by WHO as a high priority serovar [30]. Other serotypes isolated in this study, such as *S.* Agona, *S.* Vejle, and *S.* Johannesburg, are typically associated with contaminated food or environmental sources. Among these, *S.* Agona is particularly noteworthy for its emerging AMR profile. Additionally, although detected less frequently, *S.* Salamae and *S.* Johannesburg have previously been isolated predominantly from reptiles, including snakes [49,50,51].

Regarding AMR, the *Salmonella* spp. isolates obtained in our study exhibited high overall resistance 88.9% (21/24) and MDR in 50% (12/24) against the antimicrobials tested. Previous studies investigating AMR in *Salmonella* spp. have primarily focused on clinically affected animals that had received antibiotic treatment [2,15,45,52,53,54,55]. The most frequent antimicrobials for salmonellosis treatment in horses are ceftiofur, enrofloxacin, and gentamicin [15]. The use of such antibiotics may promote bacterial persistence in the gastrointestinal tract, as these drugs can disrupt the normal gut microbiota, which typically competes with *Salmonella* for nutrients and contributes to pathogen exclusion in healthy animals [15,56]. However, in this study, the highest levels of resistance were observed against sulfamethoxazole and gentamicin, with 70.8% (17/24) and 42.9% (10/24) of isolates showing resistance, respectively. This resistance profile may be considered relatively favourable, as these antimicrobials are not among the first-line drugs commonly recommended for the treatment of equine salmonellosis. Nevertheless, the frequent use of some of these agents in equine practice raises concerns regarding the potential dissemination of resistance within equine populations. The epidemiological survey carried out in the present study also considered recent treatments of the animals, of which 3.2% had received treatment. Therefore, treatment-related intestinal dysbiosis cannot explain the observed high prevalence of *Salmonella* spp. or AMR profiles. The potential association between antimicrobial treatment and *Salmonella* shedding could not be assessed in this study, as the number of horses that had recently received antibiotic therapy was not representative. According to the owners, the few treated animals had only received NSAIDs in the short to medium term prior to sampling.

Antibiotic resistance is a major concern affecting both veterinary and human medicine. Although numerous studies highlight the increasing antimicrobial resistance of *Salmonella* spp., a longitudinal study conducted by Cummings et al. (2016) [2] examined the evolution of antimicrobial resistance in *Salmonella* spp. strains over 13 years confirming a decline in resistance. As noted previously, most of these studies were conducted on symptomatic animals that had received antibiotic treatment. Nevertheless, untreated animals can still exhibit resistance due to the transmission of multidrug-resistant organisms (MDROs) between individuals or even across species boundaries [52]. This phenomenon underscores the importance of addressing antimicrobial resistance from a One Health perspective, recognizing that humans, animals, and the environment serve as interconnected reservoirs and transmission pathways for resistant microorganisms.

## 5. Conclusions

The prevalence of *Salmonella* spp. in asymptomatic horses from eastern Spain was markedly higher than previously reported in other countries, with a detection rate of 25.3%. The isolates included zoonotic serotypes of major public health relevance, such as *S.* Enteritidis, *S.* Typhimurium monophasic, and *S.* Virchow, with 50% of the isolates displaying MDR profiles or phenotypes. These findings indicate that horses may serve not only as silent reservoirs of *Salmonella* spp. but also as potential vectors for the dissemination of this pathogen and its AMR genes in shared environments, particularly where close human–animal contact occurs.

From a One Health approach, these results underscore the urgent need to implement targeted surveillance programs in equine populations, even in the absence of clinical signs, and to reinforce biosecurity protocols and antimicrobial stewardship in equine practice. Furthermore, multicenter and longitudinal studies are required to monitor temporal trends, assess geographic variability, and identify management-related risk factors, thereby advancing our understanding of the role of healthy horses in the epidemiology of *Salmonella* and the broader AMR landscape.

## Figures and Tables

**Table 1:** Temporal distribution of *Salmonella*-positive results over five consecutive days. The table includes horses with ≥1 positive sample (n = 24/95). “+”, positive culture; blank, negative. Total positives samples = 39; consecutive positives: 3 horses (2 days) and 2 horses (3 days); one horse positive on 4/5 days with an intervening negative.

POSITIVE HORSE	DAY 1	DAY 2	DAY 3	DAY 4	DAY 5
1	**+**				
2				**+**	**+**
3				**+**	**+**
4	**+**				
5	**+**	**+**		**+**	**+**
6	**+**				
7				**+**	
8		**+**			
9					**+**
10			**+**		
11	**+**		**+**		
12			**+**	**+**	
13	**+**			**+**	
14		**+**	**+**	**+**	
15		**+**		**+**	
16			**+**		
17			**+**		**+**
18			**+**		**+**
19			**+**		
20			**+**		
21			**+**	**+**	**+**
22			**+**		
23			**+**		
24		**+**			

**Table 2:** Association between epidemiological variables (sex, age, use, housing conditions, etc.) and positive *Salmonella* cultures.

Epidemiological Data	Classification in Each Group	N of Animals per Group	% of Animals Positive to *Salmonella*	*p*-Value
Age	Young	18/95	38.88 (7/18)	0.170
	Adult	55/95	18.18 (10/55)	
	Geriatric	22/95	31.81 (7/22)	
Sex	Gelding	30/95	23.3 (7/30)	0.309
	Male	29/95	17.24 (5/29)	
	Female	36/95	33.33 (12/36)	
Pathology history	Yes	10/95	20 (2/10)	0.663
	No	85/95	25 (22/85)	
Recent pharmacological treatments	Yes	3/95	0 (0/3)	<0.001
	No	92/95	26.08 (24/92)	
Horse activity	Reproduction	5/95	60 (3/5)	0.237
	Leisure	61/95	24.5 (15/61)	
	Sport	29/95	20.68 (6/29)	
Recent travel	Yes	18/95	22.22 (4/18)	0.733
	No	77/95	25.97 (20/77)	
Horse facility	Box	39/95	17.95 (7/39)	0.2
	Paddock	33/95	36.36 (12/33)	
	Both	23/95	21.73 (5/23)	
Manure storage	<10 m	18/95	50 (9/18)	<0.001 *
	11–99 m	59/95	25.42 (15/59)	
	>100 m	21/95	0 (0)	
Box cleaning frequency	Monthly	0/95	0 (0)	<0.123
	Weekly	22/95	28.57 (8/22)	
	Daily	44/95	18.18 (8/44)	
Paddock cleaning frequency	Monthly	22/95	18.18 (4/22)	<0.001 *
	Weekly	4/95	100 (4/4)	
	Daily	32/95	28.12 (9/32)	
Contact with other horses and/or animals of other species	Yes	95/95	2.4 (24/95)	
	No	0/95	0 (0)	

*Chi-square* test was used for categorical variables; *p* < 0.05 was considered significant. N = total animals sampled. * Pairwise comparisons: For manure storage, animals > 100 m differed significantly from those at 11–99 m and <10 m, with no difference between the latter two groups. For paddock cleaning frequency, weekly cleaning differed significantly from daily and monthly, with no difference between daily and monthly.

**Table 3:** Proportion of antimicrobial resistance (AMR) observed (by group and by individual antibiotic).

Antibiotic Group	Antibiotic	% AMR
Folate-pathway inhibitors	Sulfamethoxazole	70.8 ^d^ ± 10.96
	Trimethoprim	4.2 ^ab^ ± 6.88
Polymyxins	Colistin	16.7 ^ab^ ± 6.88
Quinolones	Nalidixic acid	16.7 ^ab^ ± 12.07
	Ciprofloxacin	25 ^c^ ± 12.80
Tetracyclines	Tetracycline	8.3 ^abc^ ± 9.35
Glycylcyclines	Tigecycline	16.7 ^ab^ ± 12.8
Carbapenems	Meropenem	0 ^a^ ± 0
Cephalosporins	Ceftazidime	0 ^a^ ± 0
	Cefotaxime	
Penicillins	Ampicillin	4.2 ^ab^ ± 6.88
Macrolides	Azithromycin	0 ^a^ ± 0
Amphenicols	Chloramphenicol	8.3 ^ab^ ± 6.88
Aminoglycosides	Gentamicin	42.9 ^c^ ± 13.22
	Amikacin	

^a–d^: different superscript letters within each column denote statistically significant differences (*p* ≤ 0.05) in resistance levels among the antibiotics tested. ±: standard error of the mean.

**Table 4:** Antimicrobial resistance by serotype among *Salmonella* isolates.

**Serotype**	**n**	**Folate-Pathway Inhibitors**		**Polimixins**	**Quinolones**	
		**Sulfamethoxazole**	**Trimetoprim**	**Colistin**	**1st Generation**	**2nd Generation**
					**Nalidixic Acid**	**Ciprofloxacin**
*S*. Enteritidis	11/24	4/11 (36.3%)			1/11 (9.09%)	
*S.* Johannesburg	3/24	3/3 (100%)		2/3 (66.75%)		1/3 (33.3%)
*S.* Virchow	3/24	3/3 (100%)			2/3 (66.7%)	3/3 (100%)
*S.* Typhimurium monofasic	2/24	2/2 (100%)		2/2 (100%)		1/2 (50%)
*S.* Anatum	2/24	1/2 (50%)				
*S.* Vejle	1/24	1/1 (100%)				
*S.* Salamae	1/24	1/1 (100%)				
*S*. Agona	1/24	1/1 (100%)	1/1 (100%)		1/1 (100%)	1/1 (100%)
**Serotype**	**n**	**Tetracyclines**	**Glycylcyclines**	**Carbapenems**	**Cephalosporins**	**Penicillins**
		**Tetracycline**	**Tigecycline**	**Meropenem**	**Cefotaxime**	**Ampicillin**
					**Ceftazidime**	
*S*. Enteritidis	11/24	1/11 (9.09%)	1/11 (9.09%)			
*S.* Johannesburg	3/24					
*S.* Virchow	3/24		3/3 (100%)			1/3 (33.3%)
*S.* Typhimurium monofasic	2/24					
*S.* Anatum	2/24					
*S.* Vejle	1/24					
*S.* Salamae	1/24					
*S*. Agona	1/24	1/1 (100%)	1/1 (100%)			
**Serotype**	**n**	**Macrolides**	**Amphenicols**	**Aminoglycosides**		
		**Azithromycin**	**Chloramphenicol**	**Gentamicin**	**Amikacin**	


*S*. Enteritidis	11/24		1/11 (9.09%)	4/11 (36.3%)		
*S.* Johannesburg	3/24		1/3 (33.3%)	3/3 (100%)		
*S.* Virchow	3/24					
*S.* Typhimurium monofasic	2/24			2/2 (100%)		
*S.* Anatum	2/24			1/2 (50%)		
*S.* Vejle	1/24					
*S.* Salamae	1/24					
*S*. Agona	1/24			1/1 (100%)		

For each antibiotic, cells report number resistant/number tested (percentage); blank cells indicate no resistant isolates detected. Breakpoints were interpreted according to EUCAST 2024 [32]. MIC criteria using the Thermo Scientific™ Sensititre™ EUVSEC3 plate. Antibiotic classes are folate-pathway inhibitors (sulfamethoxazole, trimethoprim), polymyxins (colistin), quinolones (nalidixic acid, ciprofloxacin), tetracyclines (tetracycline), glycylcycline (tigecycline), carbapenems (meropenem), cephalosporins (cefotaxime, ceftazidime), penicillins (ampicillin), macrolides (azithromycin), amphenicols (chloramphenicol), and aminoglycosides (gentamicin). FQ: fluoroquinolones. n denotes the number of isolates per serotype.

## Data Availability

Data are contained within the article and Supplementary Materials.

## Supplementary Materials

The following supporting information can be downloaded at: https://www.mdpi.com/article/10.3390/ani15233413/s1, Supplementary Materials S1: Field Questionnaire and Informed Consent Form for the Study of *Salmonella* spp. in Asymptomatic Horses. Supplementary Materials S2: Table S1. Antimicobials included on the EUVSEC3 Sensititre plate for EU surveillance of *Salmonella*/*E. coli* (Thermo Scientific™ Sensititre™, Madrid, Spain), showing the concentration ranges assessed and the WHO classification. These agents are recognised as of public-health importance in Decision (EU) 2020/1729.

## Abbreviations

The following abbreviations are used in this manuscript:

AMI	Amikacin
AMR	Antimicrobial Resistance
AMP	Ampicillin
ASAP	Advanced Software for Analysis of Patterns
AZI	Azithromycin
BPW	Buffered Peptone Water
CEU	Cardenal Herrera CEU University
CHL	Chloramphenicol
CIP	Ciprofloxacin
CIA	Critically Important Antimicrobial
COL	Colistin
CTA	Cefotaxime
CTZ	Ceftazidime
EUCAST	European Committee on Antimicrobial Susceptibility Testing
EUVSEC3	European Union Surveillance Sensititre Plate for Salmonella/E.Coli
FQ	Fluoroquinolone
GEN	Gentamicin
GLM	Generalized Linear Model
HIA	Highly Important Antimicrobial
HPCIA	Highest Priority Critically Important Antimicrobial
ISO	International Organization for Standardization
MDR	Multidrug Resistance
MER	Meropenem
MIC	Minimum Inhibitory Concentration
MRSA	Methicillin-Resistant Staphylococcus aureus
MSRV	Modified Rappaport Vassiliadis Agar
NAL	Nalidixic Acid
NSAID	Non-Steroidal Anti-Inflammatory Drug
NTS	Non-Typhoidal Salmonella
PCR	Polymerase Chain Reaction
PRAN	National Antimicrobial Resistance Plan (Spain)
R	Statistical Software “R”
SD	Standard Deviation
SME	Sulfamethoxazole
S.	Salmonella
TIG	Tigecycline
TET	Tetracycline
TRI	Trimethoprim
UCH-CEU	Universidad Cardenal Herrera-CEU
UELN	Universal Equine Life Number
WHO	World Health Organization
XLD	Xylose Lysine Deoxycholate Agar
