# Supplementary material for: Prevalence and Antimicrobial Resistance Patterns of Salmonella in Asymptomatic Horses in Eastern Spain: A One Health Perspective
Source: Animals (Basel). 2025 Nov 26;15(23):3413. doi: 10.3390/ani15233413 (PMC12691379; doi:10.3390/ani15233413)
Supplement: Supplementary file 1 [file animals-15-03413-s001.zip › animals-3972655-supplementary.pdf]

Supplementary Material S1. Field Questionnaire and Informed Consent Form for the Study of Salmonella spp. in Asymptomatic Horses

1. Field Questionnaire on Salmonella spp. and Antimicrobial Resistance

This questionnaire was designed to collect epidemiological, clinical, and management information from horses included in the study entitled 'Prevalence of Salmonella spp. in Asymptomatic Horses in the Valencian Community: Microbiome Changes and Antimicrobial Resistance Patterns'. The purpose of this survey is to assess potential risk factors associated with the presence of Salmonella spp. and antimicrobial resistance in the equine population.

Date of sampling: ________________________

Sample ID: ________________________

Type of sample: ________________________

Animal Data

1. Microchip number
2. UELN (Universal Equine Life Number)
3. Date of birth
4. Sex: Male / Female / Gelding
5. Breed
6. History of disease: Yes / No (If yes, specify: orthopedic, digestive, respiratory, metabolic, neurological, reproductive, or other)
7. Vaccinations: Yes / No (Specify brand)
8. Deworming: Internal / External (Specify product)
9. Recent treatments: Antibiotics / Anti-inflammatory / Other (Specify)
10. Recent travel: Yes / No (If yes, specify destination). Quarantine upon return: Yes / No
11. Primary activity: Leisure / Sport / Reproduction / Production
12. Temperament: Nervous / Calm / Aggressive / Easily stressed

Housing Data

13. Name of the riding school or facility
14. Municipality
15. District
16. Province
17. Type of housing: Outdoor / Indoor / Mixed
18. Type of construction for indoor housing: fully covered barn with internal stalls / partially covered barn / roofed stalls / concrete block walls / wooden walls / barred partitions / other (specify)
19. Type of feeding: alfalfa, hay, straw, fresh pasture, other forage (specify); compound feed, pelleted feed, alfalfa pellets, bran, beet pulp, carob, carrots, supplements, or other (specify)
20. Type of water supply: automatic drinker (constant level) / automatic drinker (push

system) / water bucket / trough with float valve / natural water source / other
21. Type of bedding: shavings (fir/pine/cypress/other), rice husk, straw, peat, sand, pellets, or other (specify)
22. Manure storage location and distance from animals: <5 m / 5–10 m / 11–30 m / 31–50 m / 51–100 m / 101–150 m / 151–200 m / >200 m
23. Cleaning frequency of facilities: stalls (daily / weekly / monthly / other), paddocks or outdoor areas (daily / weekly / monthly / other). Performed by: owner / staff / external company / other
24. Pest and rodent control: Yes / No (If yes, specify method and frequency). Performed by: owner / staff / external company / other
25. Presence of sick animals on the premises: Yes / No (If yes, specify)
26. Direct contact with other horses: Yes (same premises / external horses) / No
27. Number of horses on the premises by age: 0–3 years / 4–7 years / 8–16 years / >16 years
28. Contact with other animal species: Yes / No (If yes, specify)

Owner Information

Owner's full name: ________________________
Phone number: ________________________
Email address: ________________________

2. Informed Consent Form for Horse Owners

Project Title: 'Study on the Prevalence of Salmonella spp. in Asymptomatic Horses in the Valencian Community, Microbiome Changes, and Antimicrobial Resistance Mapping.'

Salmonellosis in horses poses a risk for both animals and humans who come into close contact with them and represents a serious concern in equine hospitals due to its infectious potential. In horses, active infection manifests with diarrhea, fever, and high mortality, and it is among the most common nosocomial infections in equine hospitals. However, some horses can act as asymptomatic carriers, as Salmonella spp. may be part of the normal intestinal flora and proliferate under conditions of intestinal dysbiosis. Determining the prevalence of asymptomatic carriers helps estimate the real risk of outbreaks in environments with high horse density (competitions, hospitals, riding schools) and assess the potential risk for people handling these animals.

Additionally, the study aims to compare the microbiome of Salmonella-positive and Salmonella-negative horses to identify risk factors associated with infection. Given the growing concern about antimicrobial resistance and its impact on animal, human, and environmental health, this project also seeks to evaluate the epidemiological status of antimicrobial resistance in the equine population.

The study objectives are:
1. To determine the prevalence of Salmonella spp. in asymptomatic horses in the Valencian Community by collecting fecal samples and performing bacterial cultures following official standardized protocols. Isolated Salmonella strains will be serotyped, and their

antimicrobial resistance profiles will be evaluated.
2. To compare the microbiome of Salmonella-positive horses with that of negative horses, by sequencing frozen fecal samples from positive animals and comparing them to negative samples from horses of similar age, diet, and management.
3. To identify the prevalence and antimicrobial resistance patterns of bacteria isolated from horses, including Escherichia coli, Staphylococcus spp., methicillin-resistant Staphylococcus aureus (MRSA), and Klebsiella spp., in accordance with the National Antimicrobial Resistance Plan (PRAN).

Sample collection:
- Five fecal samples per horse will be collected at 24-hour intervals from the rectal ampulla when possible or from freshly voided feces not contaminated by the environment.
- One nasal swab sample will be collected from both nostrils.
The sampling procedure has been reviewed and approved by the Animal Ethics Committee, ensuring animal welfare throughout the study.

Informed Consent

I, ____, born on ___________________, residing at ____, and holding ID/Passport number ___________________, acting as the legal owner of the horse named ___________________ with microchip number ___________________, DECLARE THAT:

a) I have been properly informed about:
   i. The objectives and methodology of the research project.
   ii. The role that my horse will play in this project.
   iii. The intended use of the information obtained through my horse's participation.

b) My participation is entirely voluntary and without financial compensation, and I may withdraw from the study at any time.
c) I have had the opportunity to ask questions about the study and have received and understood the relevant explanations.
d) I have full legal representation and rights over the horse.
e) I have read and understood the contents of this document, acknowledge the commitments involved, and expressly agree to participate in the study.

Therefore, as the owner and guardian of the animal, I voluntarily sign this informed consent in duplicate, expressing my willingness to participate in this study. I will receive a copy of this document for my personal records.

Signature of the Horse Owner: __________________________

Place and Date: Valencia, ____ of ____________, ______

Supplementary material S2:

Table S1. Antimicobials included on the EUVSEC3 Sensititre plate for EU surveillance of *Salmonella/E. coli* (Thermo Scientific™ Sensititre™, Madrid, Spain), showing the concentration ranges assessed and the WHO classification. These agents are recognised as of public-health importance in Deci-sion (EU) 2020/1729.

Antibiotic group	Antibiotic	Abbreviation	WHO classificaction	Concentration
Aminoglycosides	Amikacin	AMI	CIA	2 - 32 µg/mL
	Gentamicin	GEN	CIA	0.5 - 8 µg/mL
Amphenicols	Chloramphenicol	CHL	HIA	8 - 64 µg/mL
Carbapenems	Meropenem	MER	NA	0.12 - 2 µg/mL
Cephalosporins	Cefotaxime	CTA	HPCIA	0.5 - 8 µg/mL
	Ceftazidime	CTZ	HPCIA	0.5 - 8 µg/mL
Folate-pathway inhibitors	Sulfamethoxazole	SME	HIA	1 / 19 - / 152 µg/mL
	Trimethoprim	TRI	HIA	0.5 - 16 µg/mL
Glycylcyclines	Tigecycline	TIG	NA	0.5 - 4 µg/mL
Macrolides	Azithromycin	AZI	CIA	2 - 64 µg/mL
Penicillins	Ampicillin	AMP	HIA	2 - 16 µg/mL
Polymyxins	Colistin	COL	HPCIA	1 - 16 µg/mL
Quinolones	Ciprofloxacin (FQ)	CIP	HPCIA	0.12 - 1 µg/mL
	Nalidixic acid	NAL	HPCIA	>16 µg/mL
Tetracyclines	Tetracycline	TET	HIA	2 - 32 µg/mL

FQ: fluoroquinolone. Q: quinolone. WHO: World Health Organisation. HIA: highly important antimicrobial. CIA: critically important antimicrobial. HPCIAs: highest priority critical important antimicrobials. NA: not authorised for animal use. EMA: European Medicines Agency. A: avoid (by EMA categorisation). B: restrict (by EMA categorisation). C: caution (by EMA categorisation). D: prudence (EMA categorisation).
